# Smoking-induced microbial dysbiosis in health and disease

**DOI:** 10.1042/CS20220175

**Published:** 2022-09-26

**Authors:** Hagit Shapiro, Kim Goldenberg, Karina Ratiner, Eran Elinav

**Affiliations:** 1 Department of Systems Immunology, Weizmann Institute of Science, Rehovot, Israel; 2 Microbiome and Cancer Division, DKFZ, Heidelberg, Germany

**Keywords:** cancer, cardiovascular physiology, COPD, metabolites, Microbial dysbiosis, Smoking

## Abstract

Smoking is associated with an increased risk of cancer, pulmonary and cardiovascular diseases, but the precise mechanisms by which such risk is mediated remain poorly understood. Additionally, smoking can impact the oral, nasal, oropharyngeal, lung and gut microbiome composition, function, and secreted molecule repertoire. Microbiome changes induced by smoking can bear direct consequences on smoking-related illnesses. Moreover, smoking-associated dysbiosis may modulate weight gain development following smoking cessation. Here, we review the implications of cigarette smoking on microbiome community structure and function. In addition, we highlight the potential impacts of microbial dysbiosis on smoking-related diseases. We discuss challenges in studying host–microbiome interactions in the context of smoking, such as the correlations with smoking-related disease severity versus causation and mechanism. In all, understanding the microbiome’s role in the pathophysiology of smoking-related diseases may promote the development of rational therapies for smoking- and smoking cessation-related disorders, as well as assist in smoking abstinence.

## Introduction

Over 1 billion individuals worldwide smoke cigarettes, collectively generating a global health and economic burden [1]. Prolonged smoking is associated with increased morbidity, leading to an estimated average loss of a decade of life in developing countries [2–4]. Cigarette smoking increases the probability of developing cardiovascular diseases such as atherosclerosis and coronary artery disease (CAD). Smoking is considered a major driver of chronic obstructive pulmonary disease (COPD), which includes emphysema and chronic bronchitis [2,5]. Importantly, cigarette smoking enhances the risk for development of multiple cancers including lung, upper digestive tract, oral, bladder, kidney and liver cancers [5–7], by several possible mechanisms such as induction of somatic mutations and epigenetic DNA modifications [8]. Other adverse effects of smoking include increased risk of diabetes and exacerbated Crohn’s disease (CD). Smoking cessation can substantially lower smoking-related health hazards [2–4].

Multiple systemic physiological and disease-related processes can be affected by cigarette smoking, including augmented inflammatory responses in the nasal mucosa [9], airways, lung [10] and gut tissues [11,12]. In the gut, smoking modifies several functions such as mucin production [13], alterations in tight junctions in the small intestine [14], and disruption of gut barrier function [14]. In addition, cigarette smoking is associated with oxidative stress accompanied with elevated production of reactive oxygen species [10,15,16]. Collectively, these and other physiological effects of cigarette smoking may contribute to the adverse effect of smoking on health.

The microbiome is a community of microorganisms that includes bacteria, fungi, archaea, viruses and parasites, which coexist and colonize multiple mucosal niches along the mammalian host body. The microbiome can modulate host immunity, while impacting the pathogenesis of various diseases including obesity, diabetes and metabolic diseases [17–20], cardiovascular diseases [21,22], inflammatory bowel diseases [23] and neurological diseases [24,25], among others. Metagenomic sequencing of the microbiome in different body niches highlighted alterations in microbial configuration associated with a disease state [26] that are collectively termed dysbiosis. Moreover, next-generation sequencing techniques, combined with metabolomic and proteomic approaches have identified specific bacterial taxa, strains, pathways and metabolites that may be associated with human disease development and progression [26–29]. However, whether these microbial alterations drive disease or simply stem from disease conditions represents a substantial challenge in the field. As such, in humans and rodents exposed to cigarette smoke correlations were found between smoking, its pathophysiological consequences and microbial dysbiosis. It is possible that these correlations may impact smoking-related health hazards. However, in most cases, this possibility merits further mechanistic elucidation of causality.

In this review, we highlight recent advances in our understanding of the changes in the composition and function of the oral, respiratory and gastrointestinal tract microbiome induced by cigarette smoking. We discuss the possible effects of the resulting microbial dysbiosis on smoking-related diseases such as cancer, respiratory and cardiovascular diseases. We describe several smoking-related mechanisms that may correlate with the microbial dysbiosis found in health and disease. Finally, we discuss the limitations and future research directions that will help to establish the role of the microbiome in smoking-related health hazards.

## Microbial dysbiosis induced by cigarette smoking and cessation

### Oral microbiome

The oral microbiome was first identified in the late 1600s by Antonie van Leeuwenhoek, when he scraped his own dental plaque and reported ‘little living animalcules prettily moving’ under his self-constructed microscope [30]. Subsequently, many bacterial species have been cultured and attributed to diseases of the oral cavity. The oral microbiome is second only to the gut microbiome in its richness and diversity [31,32], and harbors over 700 species of bacteria [33]. Several microbial sub-habitats are included in the oral cavity including saliva, hard palate, soft palate, tongue, lip, cheek and the dental plaque biofilms on teeth, with each niche microbiome uniquely fitting to its habitat [34], and some forming a biofilm, as exemplified in the supragingival and dorsal tongue biofilms [28,35]. Disruption of the healthy oral microbiome can cause oral diseases like periodontitis and dental caries [31,36]. Furthermore, oral dysbiosis has increasingly been linked to systemic diseases of the lung, gut and cardiovascular system [37].

**Figure 1:**
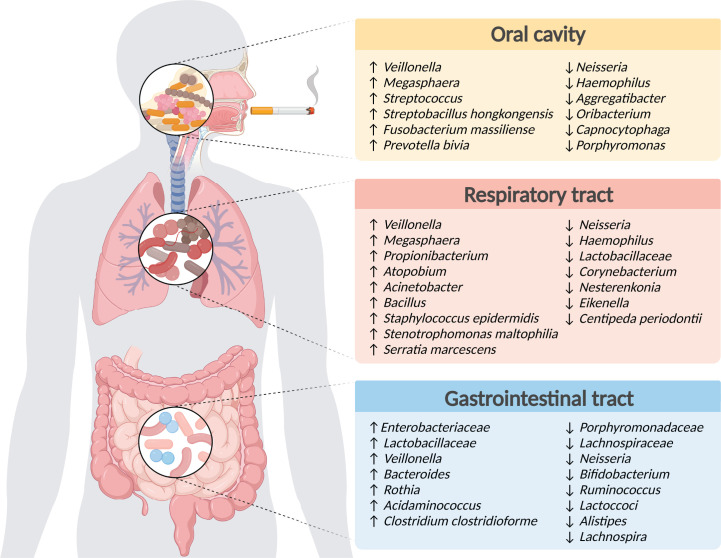
Microbial dysbiosis induced by smoking Microbial alterations reported upon exposure to cigarette smoke, in the oral cavity, respiratory and gastrointestinal tract. Figure created with BioRender (biorender.com).

Smoking may influence the microbial niches in different oral environments, change the composition of oral bacteria and can prime the mouth for colonization of pathogenic bacteria [38]. Compounds found in cigarette smoke come into direct contact with the oral microbiome, and may disrupt microbial ecology through several mechanisms, such as influencing bacterial adherence to mucosal surfaces [39], forming unstable bacterial colonization in biofilms [38], increasing the acidity of saliva, depleting oxygen [40], featuring antibiotic resistance effects [41,42] and resistance to immune cell killing by the host [40,42,43]. In a 16S rRNA gene sequencing-based study characterizing Caucasian participants with a mean age of around 70, Wu et al. [40] demonstrated that smoking caused microbial dysbiosis in oral wash samples, which included significant enrichment of *Streptococcus* and *Veillonella*, coupled with reduced abundance of *Neisseria*, *Haemophilus* and *Aggregatibacter*, in agreement with smoking-induced tongue bacterial dysbiosis in another study [44]. Smoking additionally depleted microbial aerobic metabolism pathways and led to enrichment of subgingival anerobic bacteria [40,45]. Importantly, the overall oral microbiome composition of former smokers and never smokers did not significantly differ, constituting an encouraging indication that smoking cessation can restore the healthy oral microbiome [40]. A comprehensive metagenomic sequencing of the salivary microbiome in non-smokers and smokers detected higher abundance of *Prevotella* and *Megasphaera* in smokers, whereas the genera *Oribacterium*, *Capnocytophaga*, *Porphyromonas* and *Neisseria* were significantly reduced [46]. In line with this study, lower abundance of the genus *Neisseria* was also detected in metagenomic analysis of the tongue microbiome [44] and oral rinses [47] obtained from smokers compared with individuals who never smoke. Al Bataineh et al. [48] analyzed the microbiome composition of buccal swabs from a Middle Eastern cohort of smokers using shotgun metagenomic sequencing, and found that smokers with a high nicotine dependence exhibited increased abundance of the species *Streptobacillus hongkongensis*, *Fusobacterium massiliense* and *Prevotella bivia*, which are reportedly linked to respiratory illnesses and cancer, respectively [48]. Changes in the composition of the oral microbiome may result in a variety of consequences. For example, a correlation was reported between the smoking-induced salivary microbial dysbiosis and reduced task performance network connectivity in the brain of smokers [49]. Beyond these correlative associations between smoking and oral microbial dysbiosis (Figure 1), further investigations are needed to define possible causative roles of oral microbial dysbiosis in smoking-related health hazards.

### Respiratory tract microbiome

The lung and other parts of the lower respiratory tract were once considered to be sterile, possibly due to the failure to detect microbes in the lower respiratory tract by culture-based techniques [50]. Recent advances in culture-independent microbiological techniques and the emergence of high-throughput sequencing revealed that the lung is colonized with diverse microbial communities [51].

In a small Caucasian participant-based study, Pfeiffer et al. [52] investigated the bacterial diversity along the respiratory tracts in smokers by sampling nasal and oropharyngeal swabs as well as bronchoalveolar lavage (BAL). In the nasal compartment, *Staphylococcus epidermidis*, *Prevotella* and *Propionibacterium* positively correlated with smoking, and *Corynebacterium* was negatively correlated with it. A strong overlap was noted between oropharyngeal and lung microbiota, independently of smoking status. Analysis of the oropharyngeal microbiota showed that smoking caused enrichment of the genus *Atopobium*, with reduction in the abundance of *Centipeda* and *Neisseria* [52]. In the lung, smoking led to higher abundance of the genera *Serratia marcescens* and *Stenotrophomonas maltophilia*, and reduction of *Prevotella* species [52]. Smoking may cause enrichment of biofilm forming bacteria colonizing the lung, as exemplified by *S. maltophilia*, which is capable of biofilm formation and has been implicated in lower respiratory tract infections [53]. Lim et al. [54] interrogated the association between smoking, host genetics and the microbiome in a Korean twin-family cohort using 16S rRNA sequencing of sputum samples, and found that smoking compared with genetics had the strongest effect on microbiome structure leading to a higher abundance of the genera *Veillonella* and *Megasphaera*, and a reduction in *Eikenella* and *Haemophilu*s [54]. In line with human studies, analysis of the lung microbiome of mice exposed to smoke revealed enrichment of Proteobacteria and Firmicutes phyla [55,56]. Specifically, smoking exposure led to higher abundance of the genera *Acinetobacter*, *Bacillus* and *Staphylococcus*. In mice, smoking exposure caused decreased abundance of *Oceanospirillales*, *Desulfuromonadale*s, *Nesterenkonia*, and the family *Lactobacillaceae* that were negatively correlated with proinflammatory factors such as interleukin-6 and c-reactive peptide [55,56]. In the mouse lungs, smoking exposure induced elevated inflammation and pulmonary congestion, coupled with a higher microbial diversity [55,56]. Collectively, these studies indicate that smoking leads to prominent microbial dysbiosis in the respiratory tract (Figure 1). Of note, studies exploring the respiratory microbiome need to consider the low microbial biomass of lung samples, and adequate controls need to be included to rule out common sampling contaminations that arise from the oral cavity [57].

### Gut microbiome

While almost all surfaces of the body are colonized with microorganisms, the gut harbors the highest concentration of microbial communities [58]. Exposure to cigarette smoking causes a prominent shift in gut microbiome composition in both humans and rodents. In humans who smoke, a distinct fecal composition of the microbiome was noted in comparison with the non-smoking group [59–64]. Healthy individuals who smoke compared with non-smokers exhibited enhanced abundance of fecal *Prevotella, Veillonella, Bacteroides*, *Acidaminococcus* and *Oscillospira* [60,62–64]. In addition, smokers had decreased abundance of the phylum Firmicutes [63], and more specifically depletion of the genera *Lachnospira* [64]. Smoking cessation reverted some of these microbial alterations, leading to enhanced microbial diversity, increased abundance of the phyla Firmicutes and Actinobacteria and decreased abundance of Proteobacteria and Bacteroidetes, as was shown in a longitudinal study following 4–8 weeks of cessation [59,61]. However, these longitudinal studies included small number of participants (5–10 subjects per group), and it was unclear whether the accentuated microbial alterations exist overtime with cessation and can be affected by weight gain frequently occurring during cessation.

Smoking also leads to microbial dysbiosis in other areas along the gastrointestinal tract. Shanahan et al. [65] analyzed the effect of smoking on the mucosal microbiome of the duodenum obtained from human individuals undergoing upper gastrointestinal endoscopy using 16S rRNA sequencing, and noted a lower diversity of the mucosal microbiome in participants who smoke compared with subjects who never smoke, whereas former smokers displayed an intermediate microbial composition which was in between smokers and non-smokers. The alterations in mucosal microbiome in smokers included higher abundance of Firmicutes such as *Streptococcus and Veillonella* species, enrichment of the genus *Rothia*, along with lower levels of *Neisseria and Prevotella*. Consistent with these findings, a recent study by Leite et al. [66] examined the luminal microbiome of the duodenum obtained from smokers and found a lower diversity in smokers compared with participants who never smoke. More specifically, smokers showed enrichment of *Enterobacteriaceae and Lactobacillaceae* and lower abundance of the bacterial families *Prevotellaceae, Neisseriaceae* and *Porphyromonadaceae* [66] (Figure 1). The luminal microbiome of ex-smokers was similar to that of participants who never smoke [66], which is in concert with the similarity of the fecal microbiome between ex-smokers and non-smokers [63].

Rodent studies additionally analyzed the microbial-associated communities and metabolite repertoire in different areas of the gut upon exposure to cigarette smoke [60,67–71]. Rats exposed to commercial cigarette smoke for 4 weeks had a significantly lower growth of cecal *Bifidobacterium* accompanied with lower levels of short chain fatty acids [68]. Exposure of mice to side-stream commercial cigarette smoke for 6 weeks likewise led to a shift in bacterial composition in the cecum towards enrichment of *Clostridium clostridioforme* and decreased abundance of Firmicutes phyla (mainly in *Ruminococcus* and *Lactoccoci* species), *Enterobacteriaceae* family and in segmented filamentous bacteria [69]. Moreover, chronic exposure of mice to cigarette smoke led to a significant reduction of the genus *Alistipes* in the cecum [70]. Intra-gastric exposure of mice to a smoke condensate [67] induced an increase in *Erysipelotrichaceae*, which included the genus *Allobaculum*, while reduction in *Rikenellaceae* and in the genus *Eisenbergiella*. Mice exposed to smoke condensate featured mild gut inflammation, crypt cell damage, modifications in some Paneth cell types and reduced expression of anti-microbial peptides [67]. Following infection of these mice with pathogenic bacteria, a higher susceptibility to intestinal inflammation was noted [67]. Allais et al. [13] reported that mice exposed to mainstream research cigarette smoke for 24 weeks featured microbial dysbiosis in the cecum and in the colon characterized by decreased abundance of the bacterial species *Lachnospiraceae* along with changes in intestinal mucins and expression of proinflammatory cytokines. Whether smoking-related bacterial dysbiosis induces inflammation, or alternatively smoking-induced inflammation drives dysbiosis merits further causation-seeking studies. More recent advances in multi-omics technologies such as shotgun metagenomics [71] and metabolomics improved the characterization of microbial composition, functional networks, and products caused by smoking and smoking-cessation context. These methodologies were recently used by Fluhr et al. [60], who demonstrated fecal modifications in microbial diversity, composition, metagenomic functional features and metabolites caused by smoking in both rodents and humans. Smoke exposure led to fecal microbial dysbiosis along with microbiome-dependent activation of multiple microbial metabolic pathways, such as the choline-betaine biosynthetic pathway produced by both the gut microbiome and the host resulting in increased levels of dimethylglycine (DMG), coupled with reduced N-acetylglycine (ACG). Altogether, human and rodent studies reveal prominent gut microbial dysbiosis induced by smoking exposure (Figure 1).

## Microbial dysbiosis in smoking-associated diseases

The interplay between the host and the microbiome is bilaterally affected by health status [23,26,28,37,51], and in some cases also contributes to disease development [22,25,72,73]. In the smoking context, such microbiome associations have been suggested to be implicated in several smoking-associated diseases [26,29,47,50,74]. In this section, we highlight the possible links between smoking-associated microbial dysbiosis and smoking-related diseases, such as lung cancer, colorectal cancer (CRC), COPD, CVDs, diabetes mellitus and inflammatory bowel disease (IBD), and the possible microbiome contribution to post-cessation weight gain. Of note, many of these associations still merit formal proof of causation beyond the described associations.

### COPD

COPD is a progressive obstructive airway disease that is associated with bronchitis, inflammation, emphysema and destruction of the lung parenchyma leading to limitations in airflow and gas exchange [75]. COPD is one of the leading causes of global morbidity and mortality and is directly linked to chronic exposure to cigarette smoke in the majority of cases [76]. Cigarette smoking leads to damage of airway epithelial cells, lung inflammation and impairs the airway epithelial barrier, collectively driving COPD development and exacerbation [75,77]. In COPD, the disease-associated lung microbiome deviates from healthy state in both diversity and composition [78–80]. Analysis of bronchial wash samples from COPD patients indicated an overall lower bacterial diversity and bacterial dysbiosis that were associated with COPD severity and augmented bronchial inflammation [75,81,82]. Antibiotics administration to mice lacking the airways secretory immunoglobulin A (IgA) inhibited the emphysema-like symptoms in these mice and reduced lung remodeling and inflammation, suggesting that the microbiome in COPD may have deleterious effects on disease progression [83]. Microbiome signals from other anatomical sites like the gut may also affect COPD progression [84,85].

**Figure 2:**
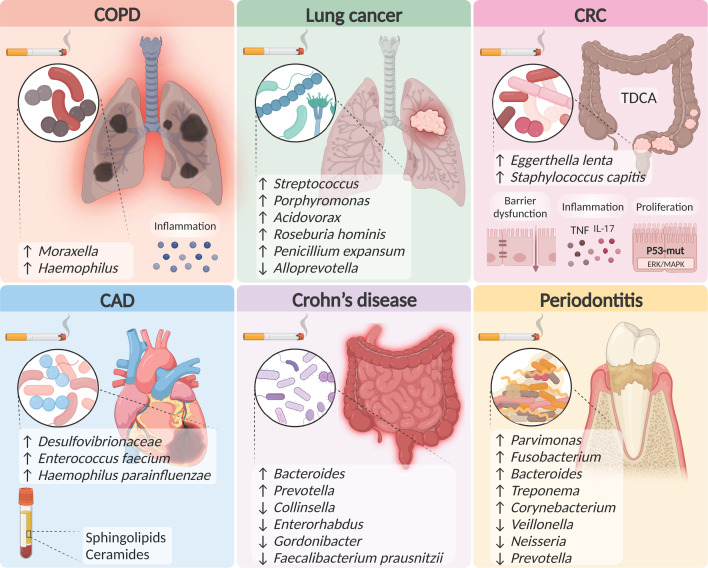
Alterations in microbial communities and metabolites in smoking-associated diseases Examples of smoking-associated microbial communities and metabolites that are modified in systemic smoking-induced diseases; Abbreviations: ERK, extracellular signal-regulated kinase; IL-17, interleukin 17; MAPK, mitogen-activated protein kinase; TNF, tumor necrosis factor. Figure created with BioRender (biorender.com).

A 16S rRNA sequencing-based analysis of the lung microbiome in healthy non-smokers, smokers and COPD patients found that smokers featured overall lower lung microbiome diversity [82,86]. COPD patients exhibited higher abundance of the phyla Firmicutes and enrichment of *Lactobacillales* [86]. However, this study enrolled a small number of participants and did not include a group of COPD patients who did not smoke, which would help to delineate the influence of smoking on the microbiome in COPD. Some of these limitations were resolved by Wang et al. [87] who found in a larger cohort that the bacterial genera *Moraxella* and *Haemophilus* were significantly enriched in ex-smokers with COPD and associated with augmented inflammation [87], in agreement with other studies correlating *Haemophilus* with COPD severity [81]. Interestingly, a study in mice [88] found that exposure of mice to smoke for 6 months followed by 3 months of cessation caused irreversible emphysema, accompanied by a lower diversity and dysbiosis of the oropharyngeal microbiome, which was reverted following smoking cessation. In the mice previously exposed to smoke, pneumococcal infection led to increased chronic lung injury along with short-term alterations in the oropharyngeal microbiome [88]. Collectively, these studies present a distinct microbiome composition induced by smoking and COPD (Figure 2). Further investigations will determine the contribution of microbial dysbiosis to COPD exacerbations, define the microbial features during smoking cessation and correlate them with COPD severity.

### Lung cancer

Lung cancer is one of the most common and deadly cancers worldwide [89]. Smoking is considered a major risk factor for lung cancer [2] and accounts for 87% of lung cancer deaths in the USA [5]. Smoking disrupts the protective epithelial barrier in the airways and interferes with cilia structures that are responsible for clearing contaminants in the lung. Cigarette smoke contains high levels of carcinogens and reactive oxygen species that can cause impairment of epithelial and endothelial function in the lung [90]. Additionally, smoking induces heterogenic DNA mutations and epigenetic modifications in bronchial epithelial cells of the lung that may result in lung carcinogenesis [8,91]. The damage to the lung tissue and to the pulmonary barrier induced by chronic cigarette smoking can allow translocation of pathogenic bacteria and increased bronchial inflammation, which altogether may contribute to lung tumorigenesis [92]. Epidemiological studies have indicated that tuberculosis infection is associated with subsequent risk of lung adenocarcinoma, and it was suggested that *Mycobacterium tuberculosis* infection may promote chronic lung inflammation and cancer development [93]. Several other clinical studies indicate that lung carcinogenesis involves dysbiosis of the lung microbiome [94–97]. Moreover, a recent study analyzed the bronchial microbiome of patients who develop lung cancer and healthy controls for 10 years and build a microbiome-based classifier, which could be used for early detection of lung cancer [95].

It is possible that the pathogenic changes caused by smoking lead to lung microbial dysbiosis, which may further contribute to lung cancer development and progression. This hypothesis was examined in the study by Lee et al. [98] who analyzed by 16S rRNA sequencing the bacterial composition of BAL samples obtained from smokers compared with non-smokers who had lung cancer. Smokers exhibited a significant increase in the ratio of Firmicutes to Bacteroidetes phyla, and more specifically had an increased abundance of *Streptococcus* and *Porphyromonas*, while the genus *Alloprevotella* was decreased [98]. Greathouse et al. [97] examined the interplay between smoking, the microbiome, p53 mutations and lung carcinogenesis and found that the genus *Acidovorax* is specifically enriched in lung tissues obtained from smokers with squamous cell carcinoma compared with patients who did not smoke. Higher abundance of *Acidovorax* was prevalent in patients with p53 mutations compared with others [97]. These findings suggest that somatic mutations together with smoking may cause a distinct microbiome composition that may contribute to lung carcinogenesis [97]. Nejman et al. [99] showed that pathways responsible for the degradation of chemicals found in cigarette smoke, such as acrylonitrile, aminobenzoates and toluene were significantly enriched in bacteria identified in lung tumors compared with other types of tumors. In addition, smoke-related metabolite degradation pathways were identified in lung bacteria obtained from the tumors of smokers. The identified lung bacteria in smokers were of the phyla Proteobacteria, Actinobacteria, and Cyanobacteria and were depleted in the Firmicutes phylum, compared with tumors of never-smokers [99]. A recent study defined the lung microbial composition in patients with non-small cell lung cancer who smoke compared with never-smokers and healthy individuals, using shotgun metagenomic sequencing of BAL samples [96]. Enhanced abundance of *Roseburia hominis*, *Pseudoalteromonas sp. CF149* and the fungus *Penicillium expansum* were found in lung cancer patients with a smoking history compared with patients with cancer who never smoke [96].

Collectively, these studies suggest that smoking leads to lung microbial dysbiosis in lung cancer patients (Figure 2). Future studies will reveal whether smoking-related microbial changes are secondary to the many microenvironmental changes characterizing lung cancer, or casually contribute to the high risk of lung cancer noted in chronic smokers. Beyond composition, studies are required to dissect microbial functional features and metabolites that are produced in response to cigarette smoke and their possible involvement in lung cancer pathogenesis. In addition to the lung microbiome, it is possible that the oral microbiome that is altered during smoking [96] may further contribute to lung tumorigenesis.

### Colorectal cancer

Smoking is also associated with an increased risk of CRC, mostly rectal cancer [100], and a longer cessation time correlates with a lower CRC risk [101,102]. Smoking is associated with genetic and epigenetic mutations leading to CRC, such as genetic microsatellite instability and mutations in oncogenes and tumor suppressor genes [103–105]. Gut dysbiosis was reported in CRC in both humans and rodent studies [106,107]. Recent studies suggested that gut microbial dysbiosis during CRC is associated with tumorigenesis, mutagenesis and epigenetic modifications related to CRC [108,109], and may potentially help in CRC detection [105,106].

Bai et al. [107] recently showed that mice treated with the carcinogen azoxymethane for CRC induction and exposed to cigarette smoke developed an increased number of colorectal tumors and enhanced proliferation of colonic epithelial cells compared with cancer-induced mice exposed to air. Notably in this study, mice exposed to smoke exhibited gut microbial dysbiosis, which included significant enrichment of *Eggerthella lenta* and *Staphylococcus capitis* and reduction in *Parabacteroides distasonis* and *Lactobacillus* species. The smoking-induced microbial dysbiosis was accompanied with elevated fecal taurodeoxycholic acid (TDCA) and other bile acids in the colon, impaired barrier function, increased activation of ERK/MAPK signaling and inflammatory IL-17 and TNF signaling pathways in colonic epithelial cells [107] (Figure 2). Germ-free mice colonized with fecal material from mice with CRC that were exposed to smoke exhibited similar gut microbial features as their corresponding donors, along with augmented proliferation of colonic epithelial cells, increased gut barrier dysfunction and elevated activation of ERK/MAPK signaling in the colon, in comparison with germ-free mice colonized with fecal material from control mice exposed to air [107]. Altogether, these results suggest a causal role for the smoking-induced microbial dysbiosis in CRC pathogenesis. It would be interesting to examine the effect of colonization of specific bacterial species or metabolites that are depleted during smoking and CRC as putative preventive or therapeutic approaches to CRC. Since prolonged smoking cessation improved survival specifically in CRC patients [110], it would be intriguing to explore the alterations in microbial features during smoking cessation and correlate them with CRC carcinogenesis. Beyond the gut microbiome, the potential changes in microbial communities outside the gastrointestinal tract during CRC is debatable [47,111] and requires future research in the smoking and non-smoking settings.

### Cardiovascular diseases

CVD constitute the leading cause of death worldwide. Smoking is considered a major CVD risk factor, accounting for over 10% of CVDs mortality [112]. Smoking cessation leads to reduced CVD [113] and mortality rate [114]. Gut microbial dysbiosis and alterations in metabolites produced by the commensal gut bacteria were found in several CVDs [115], including CAD [116,117], myocardial infarction [118], hypertension, heart failure [119] and atherosclerosis [21]. One of the most prominent examples for the effect of gut bacteria on CVD prognosis is the microbial metabolite trimethylamine (TMA) N-oxide (TMAO), which is associated with CVD risk in clinical studies [120], and promotes atherosclerosis, thrombosis and other CVDs in rodents [22]. Inhibition of TMA production by the gut bacteria caused reduction in systemic TMAO, which in turn led to attenuated atherosclerosis, and thrombosis, and improved heart function and remodeling following heart failure in preclinical murine CVD models [21,121,122].

The human association between smoking, CAD and the gut microbiome was explored by Hu et al. [123] who used 16S rRNA sequencing and metabolomics to profile the gut microbiome and serum metabolites in men with CAD who never smoke, former smokers or active smokers. In CAD patients, smokers compared with former smokers and non-smokers exhibited increased abundance of the phyla Firmicutes, and more specifically enrichment of the genus *Desulfovibrionaceae*, and the species *Enterococcus faecium, Haemophilus parainfluenzae*, coupled with a lower abundance of *Bifidobacterium catenulatum, Akkermansia muciniphila, Veillonella dispar* and *Lactobacillus johnsonii* [123]. Smokers featured elevated levels of sphingolipids and ceramides along with enhanced sphingolipids metabolism pathways [123] (Figure 2). Limitations of this study included inclusion of only men and variable cessation periods of ex-smokers. Overall, the contribution of smoking-related bacterial communities and metabolites to CAD, and the relation between resolving dysbiosis and reduced CVD risk following smoking cessation need to be further established.

Gut microbial dysbiosis has been additionally implicated in hypertension [115,119]. Wang et al. [124] explored the effects of smoking on the gut microbiome of hypertensive patients using metagenomic sequencing of fecal samples and found that smokers with hypertension had lower α‐diversity, enrichment in *Prevotella*-dominant enterotype and lower abundance of *Bacteroidetes*. These findings are in line with a previous report showing that reduced *Bacteroidetes* and increased *Prevotella* are associated with CVD risk [125]. More specifically, the genus *Phycisphaera* and the species *Clostridium asparagiforme* were depleted in smokers with hypertension compared with the other groups [124]. However, this study enrolled a small number of participants per groups. Larger study cohort may provide more information about the effect of smoking on the microbiome composition and function in hypertensive patients.

### Diabetes mellitus

Cigarette smoking increases the incidence of Type 2 diabetes [2,126]. Smoking alters glucose homeostasis by modulation of insulin sensitivity and insulin signaling, pancreatic β-cell function and exacerbation of chronic inflammation [127]. Nicotine can increase adipocyte lipolysis and loss of insulin-mediated lipolysis, leading to whole-body insulin resistance [128]. Several studies have shown that gut and oral microbial dysbiosis are associated with Type 2 diabetes [129–131], and interventional studies have used gut bacterial communities to treat diabetes and insulin resistance. One such international study included supplementation of *Akkermansia muciniphila* to obese and overweight volunteers, which resulted in improved insulin sensitivity and reduced hyperinsulinemia [72]. Other studies used the composition of the gut microbiome to predict the postprandial glucose response and to design personalized diets [20]. Ganesan et al. [132] studied the oral microbiome of Type 2 diabetes patients with periodontitis who smoke compared with non-smokers and healthy controls. In this study, all the diabetic smokers were identified with periodontitis and their oral microbiome was dominated with high levels of gram-negative facultative bacteria and significant reduction in gram negative anaerobes [132]. Future studies will determine whether oral (or possibly gut) microbial dysbiosis is merely secondary to smoking or whether it contributes to the adverse effects of smoking on diabetes and insulin resistance.

### Inflammatory bowel disease

IBD, Crohn’s disease (CD), ulcerative colitis (UC) and indeterminate colitis are a group of autoinflammatory disorders characterized by inflammation with episodes of relapse and remission that can affect different portions of the gastrointestinal tract [133]. Genetic susceptibility that includes mutations in genes associated with innate immunity and bacterial sensing along with environmental factors such as diet, stress and smoking can all contribute to IBD development and complications [133,134]. Smoking is considered a risk factor for CD [133,135], and a higher rate of CD relapse and surgical recurrence was reported in smokers compared with non-smokers, whereas smoking cessation lowered CD relapse and improved the response to therapy [136,137]. Several mechanisms have been proposed to explain the effect of smoking on CD pathogenesis, including augmented intestinal inflammation [10,67,138], epithelial cell damage and Paneth cell defects [139]. CD patients show distinct microbial composition, functional genes and metabolites compared with healthy or other IBD patients [23,62,140]. In line with these observations, *Faecalibacterium prausnitzii*, which was depleted in CD patients [141,142], showed promising results as an anti-inflammatory bacterium [142], and an anti-inflammatory protein generated by *F. prausnitzii* ameliorated colitis in mice [143]. Other approaches such as transplantation of fecal microbiome from healthy individuals to CD patients [144], and phage therapy against pathogenic bacteria associated with CD [145], are still preliminary and their effectiveness against CD remains to be determined in large clinical trials.

The combined effect of smoking and the microbiome on CD etiology has been tested in several studies. In a small study, Shahan et al. [65] analyzed the effect of smoking on the microbiome obtained from mucosa samples of the duodenum obtained from CD patients using 16S rRNA sequencing and found no effect of smoking on the upper gastrointestinal microbiome of CD patients. Using a larger cohort of CD patients, Benjamin et al. [62] found in both healthy and CD patients who were smokers a clear shift in the fecal microbiome composition compared with never smokers, which included enhanced abundance of *Bacteroides* and *Prevotella*. Likewise, a microbial signature that was specific to CD patients who smoke versus non-smokers was identified by metagenomics sequencing [146] and noted an overall reduced gut microbial diversity in CD patients, along with reduced abundance of the genera *Collinsella*, *Enterorhabdus*, and *Gordonibacter* and lower abundance of the species *F. prausnitzii* (Figure 2). Nevertheless, further studies are required to ascertain whether microbial dysbiosis is a causative or rather a result of the deleterious effect of smoking on CD. In addition to gut microbial alterations, oral microbial dysbiosis was reported in CD patients [141]; thus, it is possible that oral bacterial dysbiosis is involved in the adverse effect of smoking on CD pathogenesis. These possibilities necessitate future research.

In contrast to CD, smoking has been associated with a lower incidence and severity of UC [135]. Possible mechanisms suggested for this differential effect included modulation of small bowel inflammation, changes in immune cell populations [147], increased gut permeability [14] and other impacts induced by gut microbial dysbiosis [148]. Lo Sasso et al. [148] studied mice that were exposed to moderate levels of cigarette smoke and were subsequently induced for colon colitis development as a preclinical model for UC. The mice exposed to smoke exhibited reduced colitis severity, lower inflammatory gene expression, accompanied with compositional changes in the gut microbiome, which included enhanced abundance of the genera *Akkermansia*, *Bacteroides* and *Intestinimonas*, and reduced abundance of *Alistipes*. The impact of these smoking-associated bacterial communities on inflammation and UC severity merits further mechanistic studies.

### Diseases of the mouth

Periodontitis is highly prevalent worldwide and leads to loss of periodontal tissue structures that attach the teeth to the jawbone [149]. Smoking and diabetes are known risk factors for this disease [149]. Periodontitis, endodontic infections and dental caries are all characterized by microbial dysbiosis [32]. Mason et al. [45] found that healthy smokers had subgingival microbial dysbiosis compared with non-smokers and identified bacterial communities that were related to individuals with active dental caries. Other studies analyzed the subgingival microbiome profile from smokers and non-smokers with periodontitis and found that smokers demonstrated greater abundance of several genera such as *Parvimonas*, *Fusobacterium*, *Bacteroides*, *Treponema* and *Corynebacterium* and lower levels of some species belonging to the genera *Veillonella*, *Neisseria* and *Prevotella* [36,150] (Figure 2). These studies indicated that in patients with periodontitis, smoking leads to prominent changes in the oral microbiome composition that is associated with disease state and reduced oral health. Future longitudinal studies would help clarify the causal relationship between smoking, smoking cessation and the oral microbiome in periodontitis.

## Smoking cessation-associated weight gain

**Figure 3:**
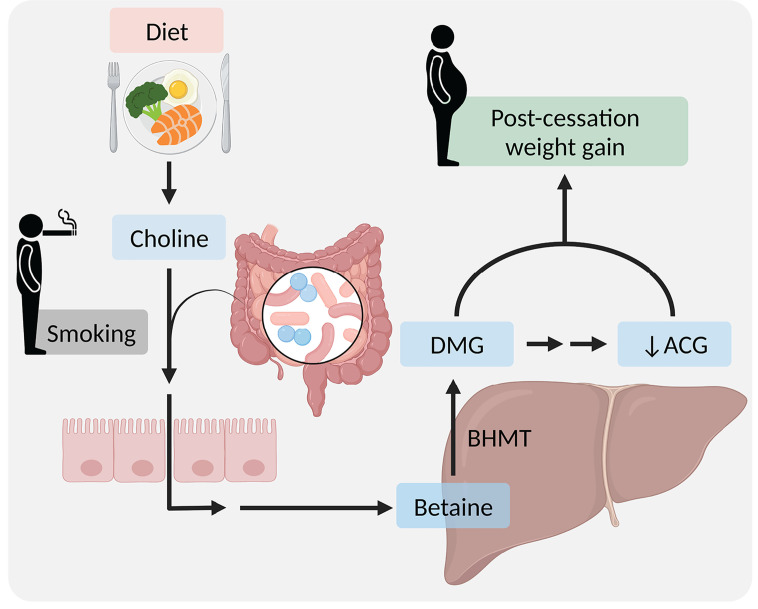
Weight gain following discontinued smoke exposure is modulated by the gut microbiome As a result of gut microbial alternations caused by smoking, the host and the gut microbiome jointly produce DMG from dietary choline and reduce ACG, which contribute to weight gain following smoking cessation. ACG, N-acetylglycine; BHMT, betaine-homocysteine S-methyltransferase; DMG, dimethylglycine. Figure created with BioRender (biorender.com).

Smoking cessation is frequently accompanied with an average weight gain of 4–5 kg within 6–12 months [151,152]. As such, post-cessation weight gain leads to low adherence to smoking abstinence [151]. Several studies reported that post-cessation weight gain did not cause significant alterations in food intake [59,153], even under conditions of low calorie intake [154]. A few studies presented a profound change in the gut microbiome composition as a result of smoking cessation accompanied with weight gain in humans and mice [59–61]. A causative role for the gut microbiome in weight gain following cessation was recently demonstrated by Fluhr et al. [60] who showed across mouse strains and diets that the post-cessation weight gain was abrogated by antibiotics and was transmissible to germ-free mice that were never exposed to smoke. The microbiome driven post-cessation weight gain was found to be involved in enhanced energy harvest and in production of DMG from dietary choline, coupled with a reduction of ACG [60] (Figure 3). DMG supplementation abolished the effect of antibiotics and led to excessive post-cessation weight gain, whereas ACG abrogated the increase in body weight. A small observational human study supported these observations [60]. Altogether, these findings indicate that the gut microbiome may contribute to post-cessation weight gain through production of bioactive metabolites that impact the host metabolic state (Figure 3). The impact of such microbial and metabolites changes on post-cessation weight gain merit further human studies and may lead to exciting interventions exploiting these checkpoints in the smoking and non-smoking metabolic settings.

## Limitations, challenges and prospects

Emerging data from human and rodent studies characterize the modified microbial composition, functional features, and metabolites observed as result of exposure to cigarette smoking. However, several limitations should be considered while interpreting these findings. Many of these studies utilize varying techniques for DNA extraction, sequencing and microbiome analysis, with most studies still rely on 16S rRNA sequencing, which offers a limited compositional resolution and nearly no functional insights into microbiome community structure. In comparison, shotgun metagenomics sequencing can identify bacterial communities at the levels of species and genus, reveal low abundance bacterial communities that can be biologically important, and detect, in high resolution, enrichment of functional genes and pathways [71]. In addition, metabolomics and proteomics analyses can uncover metabolites and proteins generated by the bacteria and the host, which may impact a variety of physiological functions. Altogether, these novel omics approaches can improve the resolution of microbial features in understanding the implications of smoking on microbiome community architecture and function.

Sampling of microbial communities and metabolites remains challenging in many human clinical studies. In gut microbiome-focused studies, fecal samples are often collected and analyzed, and assumed to represent the gut microbiome configuration, which is less accessible to sampling in most studies. However, there are important differences increasingly noted between the fecal and the microbiome residing in parts of the intestine [155]. These merit future exploration in the smoking setting. Sampling of the respiratory tract microbiome is challenged by the low microbiome biomass, which is vulnerable to contaminations and inferred sequencing. Therefore, careful adaptations in study methodologies that include DNA extraction in sterile environment, and additions of negative controls need to be included when studying such low biomass microbiome niches.

Several discrepancies between murine and human studies investigating the impact of smoking on the host–microbiome interactome merit consideration. These differences may result, for example, from exposure to different cigarettes with variable nicotine content. To overcome this limitation, in rodent studies it is recommended to use research reference cigarettes [156] with a defined amount of nicotine and other cigarette compounds. Other disparities in rodents include the smoke exposure time, rate of smoke, main-stream or side-stream exposure or even intra- gastric administration, and the degree of space sharing between smoking and control groups. The advantages of using mice models are the ability to sample different biogeographical regions of the body, define both the host and the microbiome functions and mechanisms in smoking-related diseases, and address direct causation of the microbiome, which altogether cannot be assessed in humans. However, mice models of smoke exposure are substantially limited by the fact that smoke exposure in the mouse setting (in utter contrast with humans) is an involuntary forced behavior. As such, factors related to smoking-microbiome-disease interactions that involve central nervous system contributions (such as appetite and reward behavior in human smoking cessation-induced weight gain) necessitate further exploration in human studies. In performing such human studies, one must consider variabilities arising from geographical location, gender, diet, ethnic background and weight. Longitudinal investigations of microbial alterations before and during smoking and its cessation may disentangle some of these potentially confounding factors.

It is important to note that smoking may affect bacterial communities in other niches that are distal from the primary disease site and may affect disease progression. For example, in CVDs [157], CRC [47,111] and CD [141], oral microbiome alterations were noted in addition to the gut microbial dysbiosis. Therefore, it would be intriguing to investigate the effect of smoking on the oral communities and their association with the pathophysiology of the disease. Furthermore, other organisms such as fungi, viruses and archaea are also part of the microbiome, but the impact of smoking on these organisms remains elusive. Likewise, it would be intriguing to mechanistically study in both human and rodents the possible involvement of smoking-associated dysbiosis in other smoking-related disease such as pancreatic, upper respiratory tract, upper digestive tract, bladder and liver cancer, as well as neurodegenerative disease. The disparate associations made between smoking and CD and UC can potentially involve differential pathobionts arising in patients who smoke in both disorders, and merit further studies [140,141,146,148].

In reaching an improved proof of microbial causation, one may opt to utilize microbiome depletion murine models (such as germ-free or antibiotics-treated mice) colonized with bacterial communities derived from human smokers compared with non-smokers or from mice exposed to smoke compared with mice exposed to air. Such microbiome recipients may harbor smoking-related diseases, for example COPD [83] or lung cancer [158] and will help to dissect a direct causative effect of microbiome colonization in disease development and exacerbations. Collectively, adaptation of such proposed approaches may reveal bacterial, viral, fungal members of the microbiome, and their respective metabolites that may impact smoking-associated disease pathophysiology. The contribution of bacterial metabolites to development of smoking-related disease can be assessed by administration of the candidate metabolite in mice model harboring smoking-associated disease such as COPD [83], lung cancer [158] and CRC [107]. These studies may pave the way toward incorporation of microbial interventions modifying the disease risk among smokers or optimizing their abstinence success rate.

## Open Access

Open access for this article was enabled by the participation of Weizmann Institute of Science in an all-inclusive *Read & Publish* agreement with Portland Press and the Biochemical Society under a transformative agreement with MALMAD.

## Abbreviations

ACG: N-acetylglycine
BHMT: betaine-homocysteine S-methyltransferase
CAD: coronary artery disease
CD: Crohn’s disease
COPD: chronic obstructive pulmonary disease
CRC: colorectal cancer
DMG: dimethylglycine
ERK: extracellular signal-regulated kinase
IBD: inflammatory bowel disease
IgA: immunoglobulin A
IL-17: interleukin 17
MAPK: mitogen-activated protein kinase
TDCA: taurodeoxycholic acid
TMA: trimethylamine
TMAO: trimethylamine N-oxide
TNF: tumor necrosis factor
UC: ulcerative colitis

## References

[1] Collaborators, G.B.D.T. (2021) Spatial, temporal, and demographic patterns in prevalence of smoking tobacco use and attributable disease burden in 204 countries and territories, 1990-2019: a systematic analysis from the Global Burden of Disease Study 2019. Lancet 397, 2337–2360 10.1016/S0140-6736(21)01169-7 34051883 PMC8223261

[2] Jha P. (2020) The hazards of smoking and the benefits of cessation: a critical summation of the epidemiological evidence in high-income countries. Elife 9, 1–47 10.7554/eLife.49979 PMC7093109 32207405

[3] Sakata R. et al. (2012) Impact of smoking on mortality and life expectancy in Japanese smokers: a prospective cohort study. BMJ 345, e7093 10.1136/bmj.e7093 23100333 PMC3481021

[4] Pirie K. et al. (2013) The 21st century hazards of smoking and benefits of stopping: a prospective study of one million women in the UK. Lancet 381, 133–141 10.1016/S0140-6736(12)61720-6 23107252 PMC3547248

[5] (2014) The Health Consequences of Smoking-50 Years of Progress: A Report of the Surgeon General, Atlanta (GA)

[6] Humans, I.W.G.o.t.E.o.C.R.t. (2004) Tobacco smoke and involuntary smoking. IARC Monogr. Eval. Carcinog. Risks Hum. 83, 1–1438 15285078 PMC4781536

[7] Gandini S. et al. (2008) Tobacco smoking and cancer: a meta-analysis. Int. J. Cancer 122, 155–164 10.1002/ijc.23033 17893872

[8] Alexandrov L.B. et al. (2016) Mutational signatures associated with tobacco smoking in human cancer. Science 354, 618–622 10.1126/science.aag0299 27811275 PMC6141049

[9] Jaspers I. (2014) Cigarette smoke effects on innate immune mechanisms in the nasal mucosa. Potential effects on the microbiome. Ann. Am. Thorac. Soc. 11, S38–S42 10.1513/AnnalsATS.201306-154MG 24437404

[10] Fricker M. et al. (2018) Chronic cigarette smoke exposure induces systemic hypoxia that drives intestinal dysfunction. JCI Insight 3, 1–19 10.1172/jci.insight.94040 29415878 PMC5821186

[11] Verschuere S. et al. (2011) Cigarette smoking alters epithelial apoptosis and immune composition in murine GALT. Lab. Invest. 91, 1056–1067 10.1038/labinvest.2011.74 21537330

[12] Berkowitz L. et al. (2018) Impact of cigarette smoking on the gastrointestinal tract inflammation: opposing effects in Crohn’s disease and ulcerative colitis. Front. Immunol. 9, 74 10.3389/fimmu.2018.00074 29441064 PMC5797634

[13] Allais L. et al. (2016) Chronic cigarette smoke exposure induces microbial and inflammatory shifts and mucin changes in the murine gut. Environ. Microbiol. 18, 1352–1363 10.1111/1462-2920.12934 26033517

[14] Zuo L. et al. (2014) Cigarette smoking is associated with intestinal barrier dysfunction in the small intestine but not in the large intestine of mice. J. Crohns. Colitis 8, 1710–1722 10.1016/j.crohns.2014.08.008 25205553

[15] Daijo H. et al. (2016) Cigarette smoke reversibly activates hypoxia-inducible factor 1 in a reactive oxygen species-dependent manner. Sci. Rep. 6, 34424 10.1038/srep34424 27680676 PMC5041075

[16] Messner B. and Bernhard D. (2014) Smoking and cardiovascular disease: mechanisms of endothelial dysfunction and early atherogenesis. Arterioscler. Thromb. Vasc. Biol. 34, 509–515 10.1161/ATVBAHA.113.300156 24554606

[17] Le Chatelier E. et al. (2013) Richness of human gut microbiome correlates with metabolic markers. Nature 500, 541–546 10.1038/nature12506 23985870

[18] Ley R.E. et al. (2006) Microbial ecology: human gut microbes associated with obesity. Nature 444, 1022–1023 10.1038/4441022a 17183309

[19] Turnbaugh P.J. et al. (2006) An obesity-associated gut microbiome with increased capacity for energy harvest. Nature 444, 1027–1031 10.1038/nature05414 17183312

[20] Zeevi D. et al. (2015) Personalized nutrition by prediction of glycemic responses. Cell 163, 1079–1094 10.1016/j.cell.2015.11.001 26590418

[21] Wang Z. et al. (2015) Non-lethal inhibition of gut microbial trimethylamine production for the treatment of atherosclerosis. Cell 163, 1585–1595 10.1016/j.cell.2015.11.055 26687352 PMC4871610

[22] Koeth R.A. et al. (2013) Intestinal microbiota metabolism of L-carnitine, a nutrient in red meat, promotes atherosclerosis. Nat. Med. 19, 576–585 10.1038/nm.3145 23563705 PMC3650111

[23] Lloyd-Price J. et al. (2019) Multi-omics of the gut microbial ecosystem in inflammatory bowel diseases. Nature 569, 655–662 10.1038/s41586-019-1237-9 31142855 PMC6650278

[24] Morais L.H., Schreiber H.L.t. and Mazmanian S.K. (2021) The gut microbiota-brain axis in behaviour and brain disorders. Nat. Rev. Microbiol. 19, 241–255 10.1038/s41579-020-00460-0 33093662

[25] Blacher E. et al. (2019) Potential roles of gut microbiome and metabolites in modulating ALS in mice. Nature 572, 474–480 10.1038/s41586-019-1443-5 31330533

[26] de Vos W.M. et al. (2022) Gut microbiome and health: mechanistic insights. Gut 71, 1020–1032 10.1136/gutjnl-2021-326789 35105664 PMC8995832

[27] Lynch S.V. and Pedersen O. (2016) The Human Intestinal Microbiome in Health and Disease. N. Engl. J. Med. 375, 2369–2379 10.1056/NEJMra1600266 27974040

[28] Tuganbaev T., Yoshida K. and Honda K. (2022) The effects of oral microbiota on health. Science 376, 934–936 10.1126/science.abn1890 35617380

[29] Kumpitsch C. et al. (2019) The microbiome of the upper respiratory tract in health and disease. BMC Biol. 17, 87 10.1186/s12915-019-0703-z 31699101 PMC6836414

[30] Porter J.R. (1976) Antony van Leeuwenhoek: tercentenary of his discovery of bacteria. Bacteriol. Rev. 40, 260–269 10.1128/br.40.2.260-269.1976 786250 PMC413956

[31] Human Microbiome Project, C. (2012) Structure, function and diversity of the healthy human microbiome. Nature 486, 207–214 10.1038/nature11234 22699609 PMC3564958

[32] Lamont R.J., Koo H. and Hajishengallis G. (2018) The oral microbiota: dynamic communities and host interactions. Nat. Rev. Microbiol. 16, 745–759 10.1038/s41579-018-0089-x 30301974 PMC6278837

[33] Deo P.N. and Deshmukh R. (2019) Oral microbiome: unveiling the fundamentals. J. Oral. Maxillofac Pathol. 23, 122–128 31110428 10.4103/jomfp.JOMFP_304_18 PMC6503789

[34] Xu X. et al. (2015) Oral cavity contains distinct niches with dynamic microbial communities. Environ. Microbiol. 17, 699–710 10.1111/1462-2920.12502 24800728

[35] Mark Welch J.L. et al. (2016) Biogeography of a human oral microbiome at the micron scale. Proc. Natl. Acad. Sci. U.S.A. 113, E791–E800 10.1073/pnas.1522149113 26811460 PMC4760785

[36] Shchipkova A.Y., Nagaraja H.N. and Kumar P.S. (2010) Subgingival microbial profiles of smokers with periodontitis. J. Dent. Res. 89, 1247–1253 10.1177/0022034510377203 20739702 PMC3318026

[37] Dewhirst F.E. et al. (2010) The human oral microbiome. J. Bacteriol. 192, 5002–5017 10.1128/JB.00542-10 20656903 PMC2944498

[38] Kumar P.S. et al. (2011) Tobacco smoking affects bacterial acquisition and colonization in oral biofilms. Infect. Immun. 79, 4730–4738 10.1128/IAI.05371-11 21859855 PMC3257914

[39] Brook I. (2011) The impact of smoking on oral and nasopharyngeal bacterial flora. J. Dent. Res. 90, 704–710 10.1177/0022034510391794 21558542

[40] Wu J. et al. (2016) Cigarette smoking and the oral microbiome in a large study of American adults. ISME J 10, 2435–2446 10.1038/ismej.2016.37 27015003 PMC5030690

[41] Macgregor I.D. (1989) Effects of smoking on oral ecology. A review of the literature. Clin. Prev. Dent. 11, 3–7 2689047

[42] Chien J. et al. (2020) Cigarette smoke exposure promotes virulence of pseudomonas aeruginosa and induces resistance to neutrophil killing. Infect. Immun. 88, 1–13 10.1128/IAI.00527-20 32868344 PMC7573448

[43] McEachern E.K. et al. (2015) Analysis of the effects of cigarette smoke on staphylococcal virulence phenotypes. Infect. Immun. 83, 2443–2452 10.1128/IAI.00303-15 25824841 PMC4432760

[44] Sato N. et al. (2020) Metagenomic analysis of bacterial species in tongue microbiome of current and never smokers. NPJ Biofilms Microbiomes 6, 11 10.1038/s41522-020-0121-6 32170059 PMC7069950

[45] Mason M.R. et al. (2015) The subgingival microbiome of clinically healthy current and never smokers. ISME J. 9, 268–272 10.1038/ismej.2014.114 25012901 PMC4274424

[46] Wirth R. et al. (2020) A case study of salivary microbiome in smokers and non-smokers in Hungary: analysis by shotgun metagenome sequencing. J. Oral. Microbiol. 12, 1773067 10.1080/20002297.2020.1773067 32922678 PMC7448927

[47] Kato I. et al. (2016) Oral microbiome and history of smoking and colorectal cancer. J. Epidemiol. Res. 2, 92–101 10.5430/jer.v2n2p92 28111632 PMC5241083

[48] Al Bataineh M.T. et al. (2020) Revealing oral microbiota composition and functionality associated with heavy cigarette smoking. J. Transl. Med. 18, 421 10.1186/s12967-020-02579-3 33167991 PMC7653996

[49] Lin D. et al. (2019) Association between the oral microbiome and brain resting state connectivity in smokers. Neuroimage 200, 121–131 10.1016/j.neuroimage.2019.06.023 31201984 PMC6849507

[50] Whiteside S.A., McGinniss J.E. and Collman R.G. (2021) The lung microbiome: progress and promise. J. Clin. Invest. 131, 1–1 10.1172/JCI150473 34338230 PMC8321564

[51] Wypych T.P., Wickramasinghe L.C. and Marsland B.J. (2019) The influence of the microbiome on respiratory health. Nat. Immunol. 20, 1279–1290 10.1038/s41590-019-0451-9 31501577

[52] Pfeiffer S. et al. (2022) Different responses of the oral, nasal and lung microbiomes to cigarette smoke. Thorax 77, 191–195 10.1136/thoraxjnl-2020-216153 34389656 PMC8762037

[53] Chawla K., Vishwanath S. and Gupta A. (2014) Stenotrophomonas maltophilia in lower respiratory tract infections. J. Clin. Diagn. Res. 8, DC20–DC22 10.7860/JCDR/2014/10780.5320 25653948 PMC4316254

[54] Lim M.Y. et al. (2016) Analysis of the association between host genetics, smoking, and sputum microbiota in healthy humans. Sci. Rep. 6, 23745 10.1038/srep23745 27030383 PMC4814871

[55] Li K.J. et al. (2019) Dysbiosis of lower respiratory tract microbiome are associated with inflammation and microbial function variety. Respir. Res. 20, 272 10.1186/s12931-019-1246-0 31796027 PMC6892239

[56] Zhang R. et al. (2018) Effects of smoking on the lower respiratory tract microbiome in mice. Respir. Res. 19, 253 10.1186/s12931-018-0959-9 30547792 PMC6295055

[57] Marsh R.L. et al. (2018) How low can we go? The implications of low bacterial load in respiratory microbiota studies Pneumonia (Nathan) 10, 7 10.1186/s41479-018-0051-8 30003009 PMC6033291

[58] Sender R., Fuchs S. and Milo R. (2016) Revised estimates for the number of human and bacteria cells in the body. PLoS Biol. 14, e1002533 10.1371/journal.pbio.1002533 27541692 PMC4991899

[59] Biedermann L. et al. (2013) Smoking cessation induces profound changes in the composition of the intestinal microbiota in humans. PLoS ONE 8, e59260 10.1371/journal.pone.0059260 23516617 PMC3597605

[60] Fluhr L. et al. (2021) Gut microbiota modulates weight gain in mice after discontinued smoke exposure. Nature 600, 713–719 10.1038/s41586-021-04194-8 34880502

[61] Biedermann L. et al. (2014) Smoking cessation alters intestinal microbiota: insights from quantitative investigations on human fecal samples using FISH. Inflamm. Bowel Dis. 20, 1496–1501 10.1097/MIB.0000000000000129 25072500

[62] Benjamin J.L. et al. (2012) Smokers with active Crohn’s disease have a clinically relevant dysbiosis of the gastrointestinal microbiota. Inflamm. Bowel Dis. 18, 1092–1100 10.1002/ibd.21864 22102318

[63] Lee S.H. et al. (2018) Association between cigarette smoking status and composition of gut microbiota: population-based cross-sectional study. J. Clin. Med. 7, 1–13 10.3390/jcm7090282 PMC6162563 30223529

[64] Prakash A. et al. (2021) Tobacco smoking and the fecal microbiome in a large, multi-ethnic cohort. Cancer. Epidemiol. Biomarkers Prev. 30, 1328–1335 10.1158/1055-9965.EPI-20-1417 34020999 PMC8254769

[65] Shanahan E.R. et al. (2018) Influence of cigarette smoking on the human duodenal mucosa-associated microbiota. Microbiome 6, 150 10.1186/s40168-018-0531-3 30157953 PMC6116507

[66] Leite G. et al. (2022) Smoking has disruptive effects on the small bowel luminal microbiome. Sci. Rep. 12, 6231 10.1038/s41598-022-10132-z 35422064 PMC9010470

[67] Berkowitz L. et al. (2019) Mucosal exposure to cigarette components induces intestinal inflammation and alters antimicrobial response in mice. Front. Immunol. 10, 2289 10.3389/fimmu.2019.02289 31608070 PMC6773925

[68] Tomoda K. et al. (2011) Cigarette smoke decreases organic acids levels and population of bifidobacterium in the caecum of rats. J. Toxicol. Sci. 36, 261–266 10.2131/jts.36.261 21628954

[69] Wang H. et al. (2012) Side-stream smoking reduces intestinal inflammation and increases expression of tight junction proteins. World J. Gastroenterol. 18, 2180–2187 10.3748/wjg.v18.i18.2180 22611310 PMC3351767

[70] Tam A. et al. (2020) Effects of sex and chronic cigarette smoke exposure on the mouse cecal microbiome. PLoS ONE 15, e0230932 10.1371/journal.pone.0230932 32251484 PMC7135149

[71] Durazzi F. et al. (2021) Comparison between 16S rRNA and shotgun sequencing data for the taxonomic characterization of the gut microbiota. Sci. Rep. 11, 3030 10.1038/s41598-021-82726-y 33542369 PMC7862389

[72] Depommier C. et al. (2019) Supplementation with Akkermansia muciniphila in overweight and obese human volunteers: a proof-of-concept exploratory study. Nat. Med. 25, 1096–1103 10.1038/s41591-019-0495-2 31263284 PMC6699990

[73] van Nood E. et al. (2013) Duodenal infusion of donor feces for recurrent Clostridium difficile. N. Engl. J. Med. 368, 407–415 10.1056/NEJMoa1205037 23323867

[74] Huang C. and Shi G. (2019) Smoking and microbiome in oral, airway, gut and some systemic diseases. J. Transl. Med. 17, 225 10.1186/s12967-019-1971-7 31307469 PMC6632217

[75] Decramer M., Janssens W. and Miravitlles M. (2012) Chronic obstructive pulmonary disease. Lancet 379, 1341–1351 10.1016/S0140-6736(11)60968-9 22314182 PMC7172377

[76] Burney P.G. et al. (2015) Global and regional trends in COPD mortality, 1990-2010. Eur. Respir. J. 45, 1239–1247 10.1183/09031936.00142414 25837037 PMC4531307

[77] Heijink I.H. et al. (2012) Cigarette smoke impairs airway epithelial barrier function and cell-cell contact recovery. Eur. Respir. J. 39, 419–428 10.1183/09031936.00193810 21778164

[78] Erb-Downward J.R. et al. (2011) Analysis of the lung microbiome in the “healthy” smoker and in COPD. PLoS ONE 6, e16384 10.1371/journal.pone.0016384 21364979 PMC3043049

[79] Marsland B.J. and Gollwitzer E.S. (2014) Host-microorganism interactions in lung diseases. Nat. Rev. Immunol. 14, 827–835 10.1038/nri3769 25421702

[80] Sze M.A. et al. (2012) The lung tissue microbiome in chronic obstructive pulmonary disease. Am. J. Respir. Crit. Care Med. 185, 1073–1080 10.1164/rccm.201111-2075OC 22427533 PMC3359894

[81] Mayhew D. et al. (2018) Longitudinal profiling of the lung microbiome in the AERIS study demonstrates repeatability of bacterial and eosinophilic COPD exacerbations. Thorax 73, 422–430 10.1136/thoraxjnl-2017-210408 29386298 PMC5909767

[82] Einarsson G.G. et al. (2016) Community dynamics and the lower airway microbiota in stable chronic obstructive pulmonary disease, smokers and healthy non-smokers. Thorax 71, 795–803 10.1136/thoraxjnl-2015-207235 27146202

[83] Richmond B.W. et al. (2018) Bacterial-derived neutrophilic inflammation drives lung remodeling in a mouse model of chronic obstructive pulmonary disease. Am. J. Respir. Cell Mol. Biol. 58, 736–744 10.1165/rcmb.2017-0329OC 29314863 PMC6002662

[84] Morrow J.D. et al. (2021) Peripheral blood microbial signatures in current and former smokers. Sci. Rep. 11, 19875 10.1038/s41598-021-99238-4 34615932 PMC8494912

[85] Li N. et al. (2021) Gut microbiota dysbiosis contributes to the development of chronic obstructive pulmonary disease. Respir. Res. 22, 274 10.1186/s12931-021-01872-z 34696775 PMC8543848

[86] Kim H.J. et al. (2017) The microbiome of the lung and its extracellular vesicles in nonsmokers, healthy smokers and COPD patients. Exp. Mol. Med. 49, e316 10.1038/emm.2017.7 28408748 PMC5420800

[87] Wang Z. et al. (2019) Airway host-microbiome interactions in chronic obstructive pulmonary disease. Respir. Res. 20, 113 10.1186/s12931-019-1085-z 31170986 PMC6555748

[88] Hilty M. et al. (2020) Chronic cigarette smoke exposure and pneumococcal infection induce oropharyngeal microbiota dysbiosis and contribute to long-lasting lung damage in mice. Microb. Genom. 6, 10.1099/mgen.0.000485 33295863 PMC8116676

[89] Siegel R.L. et al. (2022) Cancer statistics, 2022. CA Cancer J. Clin. 72, 7–33 10.3322/caac.21708 35020204

[90] Walser T. et al. (2008) Smoking and lung cancer: the role of inflammation. Proc. Am. Thorac. Soc. 5, 811–815 10.1513/pats.200809-100TH 19017734 PMC4080902

[91] Yoshida K. et al. (2020) Tobacco smoking and somatic mutations in human bronchial epithelium. Nature 578, 266–272 10.1038/s41586-020-1961-1 31996850 PMC7021511

[92] Jungnickel C. et al. (2015) Cigarette smoke-induced disruption of pulmonary barrier and bacterial translocation drive tumor-associated inflammation and growth. Am. J. Physiol. Lung Cell. Mol. Physiol. 309, L605–L613 10.1152/ajplung.00116.2015 26209273

[93] Liang H.Y. et al. (2009) Facts and fiction of the relationship between preexisting tuberculosis and lung cancer risk: a systematic review. Int. J. Cancer 125, 2936–2944 10.1002/ijc.24636 19521963

[94] Ramirez-Labrada A.G. et al. (2020) The influence of lung microbiota on lung carcinogenesis, immunity, and immunotherapy. Trends Cancer 6, 86–97 10.1016/j.trecan.2019.12.007 32061309

[95] Marshall E.A. et al. (2022) Distinct bronchial microbiome precedes clinical diagnosis of lung cancer. Mol. Cancer 21, 68 10.1186/s12943-022-01544-6 35255902 PMC8900294

[96] Zheng L. et al. (2021) Lung microbiome alterations in NSCLC patients. Sci. Rep. 11, 11736 10.1038/s41598-021-91195-2 34083661 PMC8175694

[97] Greathouse K.L. et al. (2018) Interaction between the microbiome and TP53 in human lung cancer. Genome Biol. 19, 123 10.1186/s13059-018-1501-6 30143034 PMC6109311

[98] Lee S.H. et al. (2016) Characterization of microbiome in bronchoalveolar lavage fluid of patients with lung cancer comparing with benign mass like lesions. Lung Cancer 102, 89–95 10.1016/j.lungcan.2016.10.016 27987594

[99] Nejman D. et al. (2020) The human tumor microbiome is composed of tumor type-specific intracellular bacteria. Science 368, 973–980 10.1126/science.aay9189 32467386 PMC7757858

[100] Hannan L.M., Jacobs E.J. and Thun M.J. (2009) The association between cigarette smoking and risk of colorectal cancer in a large prospective cohort from the United States. Cancer. Epidemiol. Biomarkers Prev. 18, 3362–3367 10.1158/1055-9965.EPI-09-0661 19959683

[101] Botteri E. et al. (2008) Smoking and colorectal cancer: a meta-analysis. JAMA 300, 2765–2778 10.1001/jama.2008.839 19088354

[102] Paskett E.D. et al. (2007) Association between cigarette smoking and colorectal cancer in the Women’s Health Initiative. J. Natl. Cancer Inst. 99, 1729–1735 10.1093/jnci/djm176 18000222

[103] Amitay E.L. et al. (2020) Smoking, alcohol consumption and colorectal cancer risk by molecular pathological subtypes and pathways. Br. J. Cancer 122, 1604–1610 10.1038/s41416-020-0803-0 32225169 PMC7250912

[104] Guinney J. et al. (2015) The consensus molecular subtypes of colorectal cancer. Nat. Med. 21, 1350–1356 10.1038/nm.3967 26457759 PMC4636487

[105] Yu J. et al. (2017) Metagenomic analysis of faecal microbiome as a tool towards targeted non-invasive biomarkers for colorectal cancer. Gut 66, 70–78 10.1136/gutjnl-2015-309800 26408641

[106] Gao R. et al. (2020) Gut microbiota dysbiosis signature is associated with the colorectal carcinogenesis sequence and improves the diagnosis of colorectal lesions. J. Gastroenterol. Hepatol. 35, 2109–2121 10.1111/jgh.15077 32337748

[107] Bai X. et al. (2022) Cigarette smoke promotes colorectal cancer through modulation of gut microbiota and related metabolites. Gut 10.1136/gutjnl-2021-325021 PMC9664112 35387878

[108] Sobhani I. et al. (2019) Colorectal cancer-associated microbiota contributes to oncogenic epigenetic signatures. Proc. Natl. Acad. Sci. U.S.A. 116, 24285–24295 10.1073/pnas.1912129116 31712445 PMC6883805

[109] Wong S.H. et al. (2017) Gavage of fecal samples from patients with colorectal cancer promotes intestinal carcinogenesis in germ-free and conventional mice. Gastroenterology 153, 1621e6–1633e6 10.1053/j.gastro.2017.08.022 28823860

[110] Ordonez-Mena J.M. et al. (2018) Impact of prediagnostic smoking and smoking cessation on colorectal cancer prognosis: a meta-analysis of individual patient data from cohorts within the CHANCES consortium. Ann. Oncol. 29, 472–483 10.1093/annonc/mdx761 29244072 PMC6075220

[111] Yang Y. et al. (2019) Prospective study of oral microbiome and colorectal cancer risk in low-income and African American populations. Int. J. Cancer 144, 2381–2389 10.1002/ijc.31941 30365870 PMC6430704

[112] Ezzati M. et al. (2005) Role of smoking in global and regional cardiovascular mortality. Circulation 112, 489–497 10.1161/CIRCULATIONAHA.104.521708 16027251

[113] Duncan M.S. et al. (2019) Association of smoking cessation with subsequent risk of cardiovascular disease. JAMA 322, 642–650 10.1001/jama.2019.10298 31429895 PMC6704757

[114] Liu G. et al. (2020) Smoking cessation and weight change in relation to cardiovascular disease incidence and mortality in people with type 2 diabetes: a population-based cohort study. Lancet Diabetes Endocrinol. 8, 125–133 10.1016/S2213-8587(19)30413-9 31924561 PMC6986932

[115] Peng J. et al. (2018) Interaction between gut microbiome and cardiovascular disease. Life Sci. 214, 153–157 10.1016/j.lfs.2018.10.063 30385177

[116] Emoto T. et al. (2016) Analysis of gut microbiota in coronary artery disease patients: a possible link between gut microbiota and coronary artery disease. J. Atheroscler. Thromb. 23, 908–921 10.5551/jat.32672 26947598 PMC7399299

[117] Senthong V. et al. (2016) Intestinal microbiota-generated metabolite trimethylamine-N-oxide and 5-year mortality risk in stable coronary artery disease: the contributory role of intestinal microbiota in a COURAGE-like patient cohort. J. Am. Heart Assoc. 5, 1–7 10.1161/JAHA.115.002816 PMC4937244 27287696

[118] Wu Z.X. et al. (2017) The changes of gut microbiota after acute myocardial infarction in rats. PLoS ONE 12, e0180717 10.1371/journal.pone.0180717 28686722 PMC5501596

[119] Marques F.Z. et al. (2017) High-fiber diet and acetate supplementation change the gut microbiota and prevent the development of hypertension and heart failure in hypertensive mice. Circulation 135, 964–977 10.1161/CIRCULATIONAHA.116.024545 27927713

[120] Tang W.H. et al. (2013) Intestinal microbial metabolism of phosphatidylcholine and cardiovascular risk. N. Engl. J. Med. 368, 1575–1584 10.1056/NEJMoa1109400 23614584 PMC3701945

[121] Organ C.L. et al. (2020) Nonlethal inhibition of gut microbial trimethylamine N-oxide production improves cardiac function and remodeling in a murine model of heart failure. J. Am. Heart Assoc. 9, e016223 10.1161/JAHA.119.016223 32390485 PMC7660847

[122] Roberts A.B. et al. (2018) Development of a gut microbe-targeted nonlethal therapeutic to inhibit thrombosis potential. Nat. Med. 24, 1407–1417 10.1038/s41591-018-0128-1 30082863 PMC6129214

[123] Hu X. et al. (2021) Impacts of cigarette smoking status on metabolomic and gut microbiota profile in male patients with coronary artery disease: a multi-omics study. Front. Cardiovasc. Med. 8, 766739 10.3389/fcvm.2021.766739 34778417 PMC8581230

[124] Wang P. et al. (2021) Cigarette smoking status alters dysbiotic gut microbes in hypertensive patients. J. Clin. Hypertens. (Greenwich) 23, 1431–1446 10.1111/jch.14298 34029428 PMC8678690

[125] Kelly T.N. et al. (2016) Gut microbiome associates with lifetime cardiovascular disease risk profile among bogalusa heart study participants. Circ. Res. 119, 956–964 10.1161/CIRCRESAHA.116.309219 27507222 PMC5045790

[126] Pan A. et al. (2015) Relation of active, passive, and quitting smoking with incident type 2 diabetes: a systematic review and meta-analysis. Lancet Diabetes Endocrinol. 3, 958–967 10.1016/S2213-8587(15)00316-2 26388413 PMC4656094

[127] Maddatu J., Anderson-Baucum E. and Evans-Molina C. (2017) Smoking and the risk of type 2 diabetes. Transl. Res. 184, 101–107 10.1016/j.trsl.2017.02.004 28336465 PMC5429867

[128] Wu Y. et al. (2015) Activation of AMPKalpha2 in adipocytes is essential for nicotine-induced insulin resistance in vivo. Nat. Med. 21, 373–382 10.1038/nm.3826 25799226 PMC4390501

[129] Qin J. et al. (2012) A metagenome-wide association study of gut microbiota in type 2 diabetes. Nature 490, 55–60 10.1038/nature11450 23023125

[130] Karlsson F.H. et al. (2013) Gut metagenome in European women with normal, impaired and diabetic glucose control. Nature 498, 99–103 10.1038/nature12198 23719380

[131] Zhou M. et al. (2013) Investigation of the effect of type 2 diabetes mellitus on subgingival plaque microbiota by high-throughput 16S rDNA pyrosequencing. PloS ONE 8, e61516 10.1371/journal.pone.0061516 23613868 PMC3632544

[132] Ganesan S.M. et al. (2017) A tale of two risks: smoking, diabetes and the subgingival microbiome. ISME J 11, 2075–2089 10.1038/ismej.2017.73 28534880 PMC5563960

[133] Torres J. et al. (2017) Crohn’s disease. Lancet 389, 1741–1755 10.1016/S0140-6736(16)31711-1 27914655

[134] Seyedian S.S., Nokhostin F. and Malamir M.D. (2019) A review of the diagnosis, prevention, and treatment methods of inflammatory bowel disease. J. Med. Life 12, 113–122 31406511 10.25122/jml-2018-0075 PMC6685307

[135] Mahid S.S. et al. (2006) Smoking and inflammatory bowel disease: a meta-analysis. Mayo Clin. Proc. 81, 1462–1471 10.4065/81.11.1462 17120402

[136] Cosnes J. et al. (2001) Smoking cessation and the course of Crohn's disease: an intervention study. Gastroenterology 120, 1093–1099 10.1053/gast.2001.23231 11266373

[137] Yamamoto T. and Keighley M.R. (2000) Smoking and disease recurrence after operation for Crohn’s disease. Br. J. Surg. 87, 398–404 10.1046/j.1365-2168.2000.01443.x 10759731

[138] Yadav P. et al. (2017) Genetic factors interact with tobacco smoke to modify risk for inflammatory bowel disease in humans and mice. Gastroenterology 153, 550–565 10.1053/j.gastro.2017.05.010 28506689 PMC5526723

[139] Liu T.C. et al. (2018) Interaction between smoking and ATG16L1T300A triggers Paneth cell defects in Crohn’s disease. J. Clin. Invest. 128, 5110–5122 10.1172/JCI120453 30137026 PMC6205411

[140] Pascal V. et al. (2017) A microbial signature for Crohn’s disease. Gut 66, 813–822 10.1136/gutjnl-2016-313235 28179361 PMC5531220

[141] Hu S. et al. (2021) Ectopic gut colonization: a metagenomic study of the oral and gut microbiome in Crohn’s disease. Gut Pathog. 13, 13 10.1186/s13099-021-00409-5 33632307 PMC7905567

[142] Sokol H. et al. (2008) Faecalibacterium prausnitzii is an anti-inflammatory commensal bacterium identified by gut microbiota analysis of Crohn’s disease patients. Proc. Natl. Acad. Sci. U.S.A. 105, 16731–16736 10.1073/pnas.0804812105 18936492 PMC2575488

[143] Quevrain E. et al. (2016) Identification of an anti-inflammatory protein from Faecalibacterium prausnitzii, a commensal bacterium deficient in Crohn’s disease. Gut 65, 415–425 10.1136/gutjnl-2014-307649 26045134 PMC5136800

[144] Fehily S.R. et al. (2021) Fecal microbiota transplantation therapy in Crohn’s disease: Systematic review. J. Gastroenterol. Hepatol. 36, 2672–2686 10.1111/jgh.15598 34169565

[145] Federici S. et al. (2022) Targeted suppression of human IBD-associated gut microbiota commensals by phage consortia for treatment of intestinal inflammation. Cell 185, 2879e24–2898e24 10.1016/j.cell.2022.07.003 35931020

[146] Opstelten J.L. et al. (2016) Gut microbial diversity is reduced in smokers with Crohn’s disease. Inflamm. Bowel Dis. 22, 2070–2077 10.1097/MIB.0000000000000875 27542127 PMC4991341

[147] Daniluk J. et al. (2017) Protective effect of cigarette smoke on the course of dextran sulfate sodium-induced colitis is accompanied by lymphocyte subpopulation changes in the blood and colon. Int. J. Colorectal Dis. 32, 1551–1559 10.1007/s00384-017-2882-9 28812128 PMC5635083

[148] Lo Sasso G. et al. (2020) The reduction of DSS-induced colitis severity in mice exposed to cigarette smoke is linked to immune modulation and microbial shifts. Sci. Rep. 10, 3829 10.1038/s41598-020-60175-3 32123204 PMC7052152

[149] Peres M.A. et al. (2019) Oral diseases: a global public health challenge. Lancet 394, 249–260 10.1016/S0140-6736(19)31146-8 31327369

[150] Moon J.H., Lee J.H. and Lee J.Y. (2015) Subgingival microbiome in smokers and non-smokers in Korean chronic periodontitis patients. Mol. Oral. Microbiol. 30, 227–241 10.1111/omi.12086 25283067

[151] Harris K.K., Zopey M. and Friedman T.C. (2016) Metabolic effects of smoking cessation. Nat. Rev. Endocrinol. 12, 299–308 10.1038/nrendo.2016.32 26939981 PMC5021526

[152] Aubin H.J. et al. (2012) Weight gain in smokers after quitting cigarettes: meta-analysis. BMJ 345, e4439 10.1136/bmj.e4439 22782848 PMC3393785

[153] Rodin J. (1987) Weight change following smoking cessation: the role of food intake and exercise. Addict. Behav. 12, 303–317 10.1016/0306-4603(87)90045-1 3687515

[154] Stamler J. et al. (1997) Relation of smoking at baseline and during trial years 1-6 to food and nutrient intakes and weight in the special intervention and usual care groups in the Multiple Risk Factor Intervention Trial. Am. J. Clin. Nutr. 65, 374S–402S 10.1093/ajcn/65.1.374S 8988949

[155] Zmora N. et al. (2018) Personalized gut mucosal colonization resistance to empiric probiotics is associated with unique host and microbiome features. Cell 174, 1388e21–1405e21 10.1016/j.cell.2018.08.041 30193112

[156] Jaccard G. et al. (2019) Mainstream smoke constituents and in vitro toxicity comparative analysis of 3R4F and 1R6F reference cigarettes. Toxicol. Rep. 6, 222–231 10.1016/j.toxrep.2019.02.009 30886823 PMC6402302

[157] Hayashi C. et al. (2011) Porphyromonas gingivalis accelerates inflammatory atherosclerosis in the innominate artery of ApoE deficient mice. Atherosclerosis 215, 52–59 10.1016/j.atherosclerosis.2010.12.009 21251656 PMC3057233

[158] Jin C. et al. (2019) Commensal microbiota promote lung cancer development via gammadelta T cells. Cell 176, 998e16–1013e16 10.1016/j.cell.2018.12.040 30712876 PMC6691977

